# Genome-wide expression patterns in physiological cardiac hypertrophy

**DOI:** 10.1186/1471-2164-11-557

**Published:** 2010-10-11

**Authors:** Ignat Drozdov, Sophia Tsoka, Christos A Ouzounis, Ajay M Shah

**Affiliations:** 1 King's College London (KCL) BHF Centre of Research Excellence - Cardiovascular Division - School of Medicine - James Black Centre - 125 Coldharbour Lane, London SE5 9NU - UK; 2 Centre for Bioinformatics - School of Physical Sciences & Engineering - King's College London (KCL) - Strand, London WC2R 2LS - UK; 3 Computational Genomics Unit, Institute of Agrobiotechnology - Centre for Research & Technology Hellas - PO Box 361, GR-57001 Thessaloniki - Greece

## Abstract

**Background:**

Genome-wide expression patterns in physiological cardiac hypertrophy. Co-expression patterns in physiological cardiac hypertrophy

**Results:**

In this study, the first large-scale analysis of publicly available genome-wide expression data of several *in vivo *murine models of physiological LVH was carried out using network analysis. On evaluating 3 million gene co-expression patterns across 141 relevant microarray experiments, it was found that physiological adaptation is an evolutionarily conserved processes involving preservation of the function of cytochrome c oxidase, induction of autophagy compatible with cell survival, and coordinated regulation of angiogenesis.

**Conclusion:**

This analysis not only identifies known biological pathways involved in physiological LVH, but also offers novel insights into the molecular basis of this phenotype by identifying key networks of co-expressed genes, as well as their topological and functional properties, using relevant high-quality microarray experiments and network inference.

## Background

Physiological left ventricular hypertrophy (LVH) is a complex cardiac adaptive response to chronic exercise [1], sometimes referred to as the "athletic heart" [2]. It is characterized by an increase in left ventricular (LV) mass, wall thickness and chamber size, underpinned by profound molecular and biochemical changes, that allows the heart to efficiently provide an increased cardiac output during periods of exercise [1]. The physiological LVH state can typically be maintained for months or years without significant compromise of cardiac function. In contrast, pathological LVH occurring in response to chronic cardiac overload, imposed by diseases such as hypertension, is characterized by a progression to contractile dysfunction and heart failure and an increased long-term mortality [3]. Other differences between physiological and pathological LVH include the occurrence of significant fibrosis and capillary rarefaction in the latter condition. Due to the stark clinical contrast between physiological and pathological LV remodeling, it is of importance to delineate the precise molecular mechanisms that drive these divergent responses to stress.

Some progress has been made in elucidating mechanisms of physiological hypertrophy through a number of genomic analyses and several reports implicate activation of the phosphoinositide-3-kinase (PI3K)/Akt pathway as an important component [1]. More recent studies offer the possibility to examine gene expression patterns in this phenotype more consistently and broadly [4,5]. However, restrictions still exist, primarily due to an innate heterogeneity of signaling cascades and limitations of conventional statistical methods to address higher order relationships between genes. Visualization and analysis of biological data as networks is a powerful explorative alternative with the capacity to accurately assess complex relationships and eliminate noise inherent to microarray experiments [6]. Although such methods have already been successful in defining miRNA signature in obesity and diabetes [7], discovering novel cancer-associated genes [8], and predicting the involvement of genes in core biological processes [9], their use in cardiovascular biology has been limited [10].

Recent availability of comprehensive mouse cardiac hypertrophy microarray datasets, deposited in resources such as ArrayExpress [11] and Gene Expression Omnibus [12], makes it possible to investigate global molecular mechanisms of this phenotype. The inference of gene relevance networks by co-expression analysis is based on the hypothesis that genes encoding proteins participating in the same pathway or biological process may often be co-regulated under a large number of experimental conditions [13]. An important advantage of network analysis algorithms is their ability to exploit local structure between biologically related nodes, thus eliminating most of the inherent noise [6]. Additionally, confidence in network inference through co-expression analysis may be increased by an integrative approach that utilizes multiple datasets across a variety of experimental conditions and microarray platforms [14].

In this study, a computational approach has been undertaken that identifies key expression patterns of physiological LVH using integrative analysis of 3 million gene co-expressions across 141 relevant microarray conditions. We included transcriptome data from studies in mouse models of physiological LVH induced by swimming exercise, cardiac-specific activation of Akt, and cardiac-specific activation of PI3K. This is the first study in cardiac hypertrophy at this scale and it may provide a basis for further understanding of both physiological and pathological LVH phenotypes.

## Results

### Generation of Microarray co-expression Networks

Gene expression profiles in heart tissue were investigated under normal conditions, during physiological (exercise) stress, and in two gene-modified models of physiological LVH involving cardiac activation of the PI3K/Akt pathway. To estimate the specificity of the hypertrophic gene signature, an additional dataset monitoring gene expression in healthy mouse organs was also used.

Four mouse microarray datasets totaling 141 arrays were obtained from ArrayExpress for further analysis (Table 1). The Akt dataset was generated using a tetracycline-regulated transgenic system with the capacity to conditionally switch a constitutively active form of the Akt1 protein kinase on or off in the adult heart [15]. This dataset consisted of normal heart tissue (*n *= 4), short-term (2 weeks), activated Akt1 (*n *= 4), and switched-off Akt1 (2 days following a 2 week activation, *n *= 4). The PI3K dataset consisted of wild type hearts (*n *= 3) and hearts with expression of dominant-negative PI3K (*n *= 3) or constitutively active PI3K (n = 3) [16]. The Swimming dataset, containing 30 arrays, monitored expression in mouse hearts under normal conditions, swimming (short- and long-term), and swimming followed by 1 week of rest [17]. Finally, the Normal dataset (*n *= 90 arrays) monitored transcript expression in healthy mouse tissues including bladder, bone, spleen, stomach, and the heart [18].

**Table 1:** Summary of microarray datasets included in the analysis.

Dataset Name	Experimental Condition	ArrayExpress Accession	Platform	No. of Probes	Normalized No. of Probes
Akt	Wild Type, Short-term Akt1 induction (n = 12)	E-GEOD-3383[15]	Mouse430A_2	~34,000	12127

PI3K	Wild Type, caPI3K, dnPI3K (n = 9)	E-GEOD-558[16]	MG-U74A	~12,000	7511

Swimming	Wild Type, Short-term exercise, long-term exercise (n = 30)	E-GEOD-77[17]	MG-U74A	~12,000	7511

Normal	Healthy tissue (n = 90)	E-GEOD-97	MG-U74A	~12,000	7511

caPI3K = constitutively active form of PI3K, dn = dominant-negative form of PI3K.

**Figure 1:**
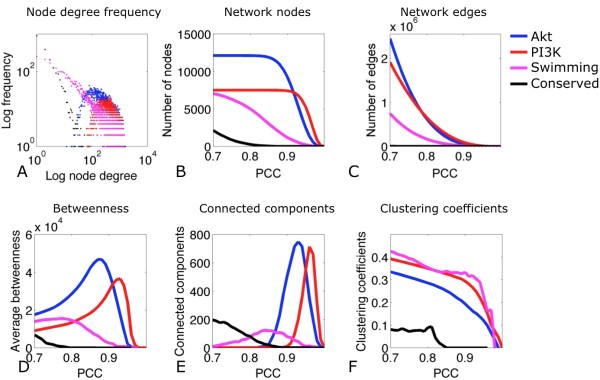
**Properties of gene co-expression networks**. **(1A) **Log-log plot of node degree and frequency distribution suggests that all microarray networks and their intersection - the Conserved network - are scale-free. **(1B) **Relationship between gene inclusion in the networks as a function of Pearson correlation coefficient (PCC). **(1C) **Relationship between the number of co-expression links (edges) as a function of PCC. **(1D) **Average network betweenness centrality as a function of PCC. **(1E) **Connected components as a function of PCC: at large PCC values, networks had a tendency to break down into a large number of connected components, i.e. unconnected subgraphs. **(1F) **Clustering coefficients as a function of PCC: as PCC threshold increased, genes within each subgraph lost the tendency to cluster together, a property reflected by the average clustering coefficient.

After pre-processing (see **Methods**), pair wise gene expression similarities were measured using the Pearson Correlation Coefficient (PCC). Co-expression networks were "undirected" and, at PCC ≥ 0.70, obeyed a power law, suggesting a scale-free architecture dominated by a number of highly connected "hub genes" (Figure 1A). The PCC threshold was set to 0.70 on the basis of the following evidence: (i) gene correlation profiles with PCC over 0.60 were demonstrated to be more biologically relevant [19] and (ii) similar studies of human gene co-expression landscape [20] have employed comparable threshold criteria. Additionally, below this cut-off all networks were excessively large (average node degree >500), suggesting a presence of false-positive edges. However, a more stringent PCC threshold was avoided, as further filtering has been implemented by selecting gene pairs that were correlated across all three datasets. Finally, the "data driven cut-off" approach (as performed by [19]) was not deemed appropriate as it is intended primarily for comparison of multiple networks derived from differential phenotypes.

At PCC ≥ 0.70 it was noted that an increase of this cut-off value removed weakly connected links from all networks while maintaining a constant number of genes (Figure 1B, C). Average betweenness centrality (see **Methods**) of all networks increased with PCC values, suggesting that applying a threshold to each network removed peripheral nodes and edges, leaving critical hubs intact (Figure 1D). Additionally, increasing the PCC threshold resulted in fragmentation of networks into a large number of structured subgraphs, reflected in the number of connected components and clustering coefficients (Figure 1E, F). Overall, networks derived from hypertrophic tissues were highly structured (Table 2), characterized by nodes with multiple connections (average degree range: 211.3 to 508.6), small network diameters (range: 3.0 to 5.9) and relatively high clustering coefficients (range: 0.35 to 0.40).

**Table 2:** Network Statistics at PCC = 0.70 for mouse microarray networks and the Conserved network.

Dataset	Number of Genes	Number of Interactions	Avg. Degree	Network Diameter	Clustering Coefficient
Akt	12127	2446804	403.5	3.3	0.35

PI3K	7511	1910082	508.6	3.0	0.39

Swimming	7047	744666	211.3	5.9	0.40

Conserved	2128	4144	3.9	11.8	0.10

Normal	3983	91544	45.9	4.58	0.53

### Co-expression model of Physiological cardiac hypertrophy

**Figure 2:**
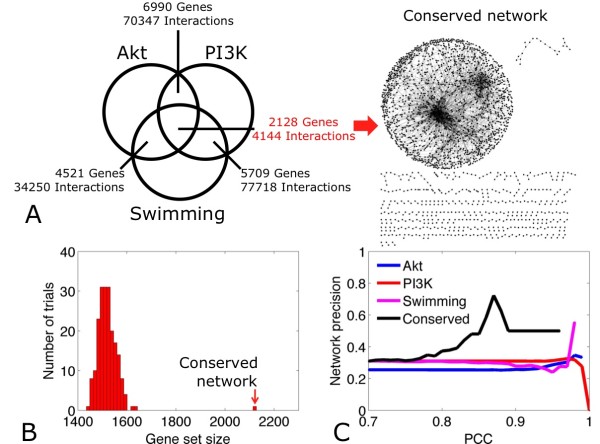
**Co-expression model of physiological hypertrophy**. **(2A) **Venn diagram of intersection between Akt, PI3K, and Swimming microarray networks (left) yielding a Conserved network with 2128 genes (represented by nodes) and 4144 co-expressions (represented by lines) (right). **(2B) **Distribution of the co-expression intersection for randomly generated networks, compared with the Conserved network at PCC = 0.70. **(2C) **Network precision, defined as the proportion of genes that mapped to the KEGG pathway database, for microarray datasets and the Conserved network.

Due to the large number of genes and co-expression links observed in this analysis, some observations could be due to experimental artifacts and thus of questionable biological relevance. The recurrence of a co-expression link in all three microarray datasets was considered to increase the reliability of the inference. At PCC = 0.70, the Akt and PI3K networks shared 6990 genes and 70347 interactions, the PI3K and Swimming networks shared 5709 genes and 77718 interactions, and the Akt and Swimming networks shared 4521 genes and 34250 interactions. There were 2128 genes and 4144 interactions common to all three networks, which formed a consensus 'Conserved' gene co-expression network (Figure 2A, Additional files 1 and 2). Similarly to the Akt, PI3K, and Swimming networks, the Conserved network was scale-free.

To evaluate the statistical significance of the Conserved network, three randomized networks were generated. Randomization was performed by shuffling edges of the Akt, PI3K, and Swimming networks 4× (number of edges) times, while preserving the node degrees of the original networks [21] This procedure was repeated 200 times (see **Methods**). The simulation showed that on average, the three random networks shared 1519 co-expressed genes (standard deviation = 35) and that at most their intersection contained 1641 genes (Figure 2B). These results indicated that identification of 2128 genes in the Conserved network is statistically significant (z-score = 17.1).

Phenotype specificity of the Conserved network was estimated by comparing it to gene co-expressions inferred from the Normal mouse transcriptome (3983 genes, 91544 interactions; PCC≥0.70) [18]. It was hypothesized that the extent of conserved nodes and edges between two networks may correspond to molecular mechanisms shared by the LVH phenotype and cells under basal conditions. Interestingly, it was determined that the Conserved and Normal networks shared only 88 genes and 57 co-expressions, confirming that the Conserved network may reflect LVH-specific cardiac response.

To gauge the extent of validated molecular pathways in all co-expression networks, all genes were mapped to the KEGG pathway database [22]. Genes with annotations in KEGG pathways were considered to be true positives and network precision (specificity) was estimated as the proportion of true positive genes to the overall number of genes in a network (true positive cases plus presumed false positives). At PCC = 0.70, network precision for the Akt, PI3K, Swimming, and Conserved networks approached 31%. Interestingly, it was noted that while increasing PCC threshold had no apparent effect on specificity of individual microarray networks, specificity of the Conserved network increased up to two-fold with PCC values (Figure 2C), suggesting that gene pairs with high PCCs in the Conserved network are likely to be well-annotated molecular entities.

**Figure 3:**
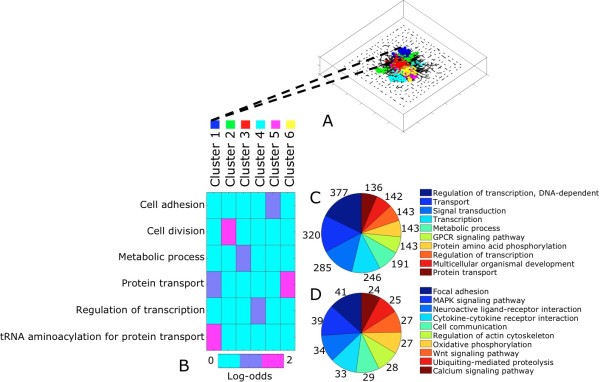
**Functional interpretation of the Conserved network**. **(3A) **Network visualization of top 6 MCL clusters in the Conserved network. Each node is a gene, color-coded according to MCL cluster membership. **(3B) **Functional enrichment of MCL clusters in the Conserved network. Clusters were enriched for Gene Ontology Biological Process (GO-BP) terms and strength of enrichment was measured by the log-odds ratio (see **Methods**). The top over-represented term for each cluster is listed. **(3C) **Frequency distribution of top 10 Gene Ontology Biological Process (GO-BP) terms across all genes in the Conserved network. **(3D) **Frequency distribution of top 10 KEGG Pathways terms across all genes in the Conserved network.

All genes in the Conserved network were processed further by MCL clustering (inflation parameter = 1.3) (see **Methods**). There were 302 clusters, of which six contained >40 genes. The largest cluster consisted of 245 genes (Figure 3A). Enrichment of each MCL cluster for GO Biological Process (BP) terms (see **Methods**) identified processes such as 'tRNA aminoacylation for protein transport' (Cluster 1), 'Cell division' (Cluster 2), and 'Protein transport' (Cluster 6) (Figure 3B). At the gene level, the Conserved network was representative of GO-BP terms such as 'Regulation of transcription' (*n *= 377 genes), 'Transport' (*n *= 320 genes), and 'Signal transduction' (*n *= 285 genes), as well as KEGG pathways such as 'Focal adhesion' (*n *= 41 genes), 'MAPK signaling pathway' (*n *= 39 genes), and 'Neuroactive ligand-receptor interaction' (*n *= 24 genes) (Figure 3C, D).

The generation of a Conserved network for physiological cardiac hypertrophy consisting of 2128 genes (Additional file 1) and 4144 interactions (Additional file 2), based on a series of relevant microarray experiments and computational processing of gene expression similarities, is thus a first step towards the discovery of the molecular underpinnings of this phenotype, its basic components and their structural and functional features.

### Identification of Critical Hubs in the Conserved co-expression Network

**Figure 4:**
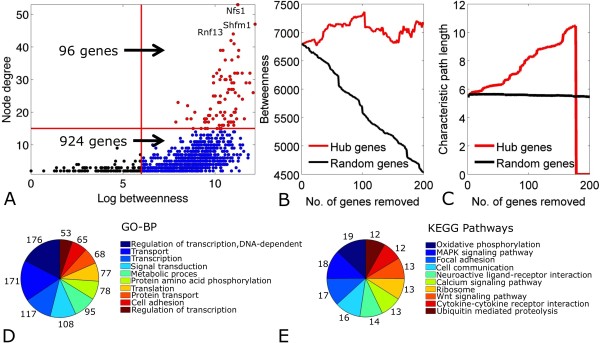
**Core network in physiological cardiac hypertrophy derived from co-expression network topology**. **(4A) **1020/2128 topologically-central genes in the Conserved network formed the Core network. 96 genes had large betweenness centrality and node degree values (red), while 924 genes had only large betweenness centrality values (blue). Most connected genes were Nfs1, Shfm1, and Rnf13, as labeled. **(4B) **Betweenness centrality of the Conserved network was not sensitive to the systematic removal of top 200 genes with greatest node degree compared to randomly selected genes. **(4C) **Characteristic path length in the Conserved network increased exponentially following removal of 200 most connected genes compared to the removal of 200 randomly chosen genes. **(4D) **Top 10 over-represented Gene Ontology Biological Process (GO-BP) terms and KEGG Pathways terms across 1020 core genes.

The topology of the Conserved network was explored further to identify hub genes. Betweenness centrality (relative importance of a gene within a graph) and node degree (number of co-expression links for a gene) were measured for 2128 genes (Figure 4A). There were 1020 genes (48% of 2128) with high betweenness centrality (log betweenness centrality > 6), connected by 3047 interactions (74% of 4144). These 1020 genes formed the "core" of the Conserved network (defined as the Core network) mainly because changes in their expression and/or structure are likely to alter behavior and topology of the overall network. Remarkably, 96 out of 1020 genes had both high betweenness centrality and node degrees (log betweenness centrality > 6 and node degree > 15). These genes tended to localize at the center of the network, while the other 924 genes aligned along the periphery (Figure 4B). The three genes with the greatest values for both topological parameters were Nfs1 (nitrogen fixation 1 homolog), Shfm1 (deleted in split hand/split foot protein 1), and Rnf13 (ring finger protein 13). It is interesting to note that Nfs1 is an aminotransferase [23] with a cysteine desulfurase function [24] implicated in Freidrich's ataxia [25], a complex disease often associated with a hypertrophic cardiomyopathy phenotype [26]. Furthermore, Shfm1 is the gene most likely associated with Split hand/split foot malformation (SHFM) in region 7q21.3-q22.1 [27], a disease exhibiting congenital heart defect phenotypes [28]. Finally, Rnf13 is a trans-membrane RING-type E3 ubiquitin ligase highly expressed in pancreatic ductal adenocarcinoma [29], but also expressed in chicken embryo brain and heart [30]. It follows that most of the other 96 genes uncovered by using the above mentioned topological parameters might also be implicated in expression patterns with a phenotype associated with heart tissue.

To test the hypothesis that hub genes may be crucial to the overall structure of the discovered network, the 200 most connected genes were systematically removed from the network. To assess network integrity, average betweenness and characteristic path length (see **Methods**) were measured. Betweenness did not change drastically following systematic removal of the top connected nodes compared to random node removal (Figure 4C). However, systematic removal of hubs increased characteristic path length to a threshold beyond which it rapidly collapsed due to splintering of the core network into small subnetworks. Characteristic path length was unaffected by removal of random genes and the network remained intact (Figure 4D).

It was then of interest to identify biological processes represented by the core network. Of 1020 core genes, 176 participated in 'DNA-dependent regulation of transcription', 171 in 'Transport', and 117 in 'Transcription'. Additionally, the 1020 genes were mapped to KEGG pathways such as 'Oxidative phosphorylation' (*n *= 19 genes), 'MAPK signaling pathway (*n *= 18 genes), and 'Focal adhesion' (*n *= 17 genes) (Figure 4E). Evidently, not all genes can be associated with GO or KEGG classes.

**Figure 5:**
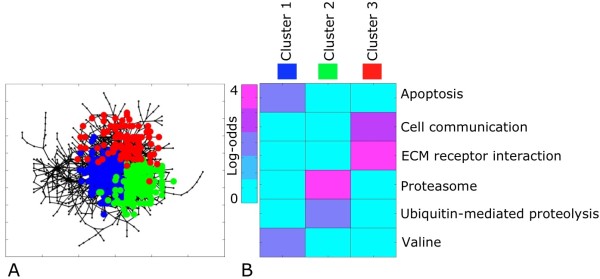
**Clustering and KEGG pathways enrichment of the "core" conserved network**. **(5A) **MCL clustering of the core gene network identified 3 major clusters, each represented by a unique color **(5B) **Heatmap of gene counts in the core network clusters 1-3, enriched for KEGG Pathway terms (log-odds ratio enrichment, see **Methods**). The top over-represented term for each cluster is listed.

The topology of the core network was further interrogated by MCL clustering (inflation parameter = 1.3). MCL partitioned the core network into 48 clusters. The largest cluster contained 252 genes. Overall, there were 7 clusters with 20 or more genes, representing 70% of the core network (Figure 5A). Because each cluster may contain genes involved in a common molecular pathway, over-represented KEGG pathways for each cluster were identified using the log-odds ratio (see **Methods**). Only the largest three clusters could be detected as enriched by KEGG pathways, due to low counts. For example, Clusters 1 was mostly representative of 'Apoptosis' and 'Valine, leucine, and isoleucine degradation' (n = 8 genes and n = 4 genes respectively) while cluster 2 was representative of 'Proteasome' (*n *= 6 genes). (Figure 5B).

**Figure 6:**
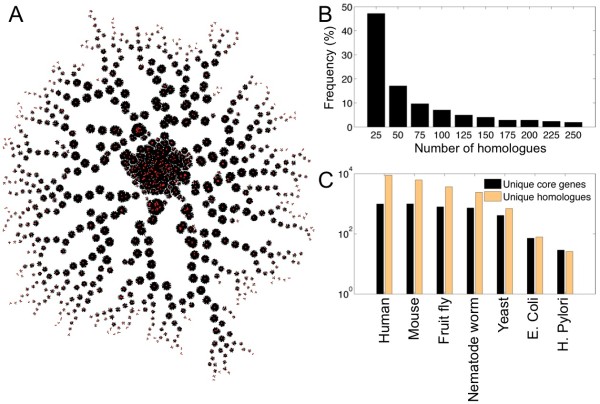
**Evolutionary properties of the core genes in physiological hypertrophy**. **(6A) **Network visualization of the core protein homology network produced by best BLAST hits across full genome sequences of 287 species in the COGENT database. Red nodes represent the core proteins, while the black edges represents homologous relationships. **(6B) **Frequency distribution of the core gene homologues. **(6C) **Cross-species conservation of core hypertrophic proteins.

The question arises whether the 1020 genes of the Core network are also evolutionarily conserved. These genes were compared against the complete genomes of 287 species stored in the COGENT database (see **Methods**), resulting in a network of 100532 pairwise sequence similarities covering 64550 unique homologues (Figure 6A, Additional file 3). There are 271 genes that match 200 species or more, while the frequency distribution of core gene homologues is a typical distribution for sequence similarities (Figure 6B) [31]. Only 7 genes do not have a homologue apart from human or mouse (Ensembl Mouse Ids: 00000027596, 00000031329, 00000039153, 00000045107, 00000061555, 00000078135, 00000079523), most of them encoding proteins of unknown function, except 00000078135 which encodes the 'EP300 interacting inhibitor of differentiation 1' gene. The following numbers of core genes have detected a number of unique homologues, respectively as follows: 993 detect 8928 in human, 999 detect 6235 in mouse, 794 detect 3697 in *Drosophila melanogaster*, 728 detect 2424 in *Caenorhabditis elegans*, 413 detect 697 in *Saccharomyces cerevisiae *S28, just 72 detect 79 in *Escherichia coli *and 29 detect 26 in *Helicobacter pylori *J99 strain (Figure 6C), strongly indicating that the majority of the detected genes are confined to the mammalian taxonomic range. The high numbers for the animal model species as well as mouse and human are derived from extensive paralogous families within this set. Further investigation is necessary to understand the evolutionary history of the detected core network.

Thus, the generation of a core network for physiological cardiac hypertrophy reduces the initial number of genes to just over a thousand and consequently allows the further study of a more compact dataset, based on topological feature detection. The discovery of both known and newly detected cases in terms of genes and gene sets, along with their functional and evolutionary properties represents a consolidation of information that can be obtained from multiple microarray experiments for this key phenotype.

## Discussion

Physiological stimuli such as chronic exercise lead to compensatory growth and remodeling of the heart associated with preserved or improved cardiac function. Recently, class IA phosphoinositide 3-kinase (PI3K) and Akt1 have emerged as important regulators of physiological adaptation [32,33] but the broader signaling cascades associated with physiological LVH remain poorly understood. In this study we show that network analysis has the potential to infer genome-wide biological mechanisms related to physiological LVH phenotype. Importantly, we report on the network topology and functional properties of the physiological LVH networks, the first such analysis in a mammalian cardiovascular system.

Gene expression profiles were used to identify conserved gene co-expression patterns in PI3K, Akt1, and Swimming models of physiological LVH and to obtain a global overview of biological functions involved in physiological cardiac remodeling. Previous reports have explored gene co-expression networks derived from heterogeneous microarray platforms [14,34] and confirm that observing a conserved gene co-expression suggests a biological relevance [9,35]. The consensus gene co-expression model, referred to as the Conserved network, consisted of 2128 genes and 4144 links (Additional files 1 and 2). It was confirmed to be scale-free, highly structured, and non-random, suggesting the presence of a small number of critical hub genes that may be biologically relevant. Additionally, the Conserved network had only a trivial intersection with the Normal interactome (88 genes, 57 links), suggesting that our consensus model may present a reliable physiological LVH signature. Topological features were consistent with the general behavior of biological networks [36] and topologies detected in protein-protein interaction collections such as STRING [37]. At PCC≥0.70, 31% of all genes in the Conserved network were identified in the KEGG pathways database. This coverage increased exponentially with PCC threshold, approaching 80% at PCC = 0.88 (Figure 2C). These results are comparable to previous studies of co-expression networks [20] and suggest that an increase in PCC stringency produces a marked positive effect on network precision.

Due to a large number of co-expression links (3 million), it is possible that some of these links are artifacts or byproducts of systematic error. Thus, evaluation of conserved co-expression links across three physiological LVH networks has a number of strengths compared to conventional statistical approaches. First, reproducible co-expressions are less likely to be false-positives and may reflect biologically relevant links, thus presenting a reliable interactome for further experimental validation [9,38]. For example, in a recent meta-analysis of >300 tissue samples of gastric cancer, this hypothesis helped to identify a functional link between prognostic marker PLA2G2A and the EphB2 receptor [34]. Second, network intersections account for putative platform- or experiment-dependent variability (e.g. number of transcripts) between multiple microarray datasets [14]. Third, due to the heterogeneous (molecular and physiological) nature of physiological LVH models, conserved co-expressions provide an overview of common regulatory mechanisms.

These assumptions were confirmed using automated PubMed queries, whereby each gene in the Conserved network was searched in the context of 'hypertrophy', 'heart', or 'heart failure'. Indeed, 933 out of 2128 (44%) genes in the Conserved network had at least one abstract per search term while 50 of those have at least one hundred abstracts for all terms, suggesting that the Conserved network provides an acceptable coverage of current molecular knowledge of cardiac biology (Additional file 4).

The Conserved network may be used to describe the regulatory mechanisms underpinning the cardiac remodeling response to physiological stress. 'Oxidative phosphorylation' was noted as one of the most abundant KEGG pathways (*n *= 27 genes). The most over-represented members of this pathway were genes encoding subunits of mitochondrial cytochrome c oxidase (COX) (*n *= 6 genes). COX is localized to the inner membrane of mitochondria and is the last component of respiratory chain. To sustain respiration, this enzyme catalyzes the transfer of electrons from cytochrome c to molecular oxygen and facilitates the aerobic production of ATP by ATPsynthase (*n *= 2 genes in the Conserved network) [39]. To maintain efficient cardiac contractility under increased energetic demand, the regulation of COX function must be preserved. In post-myocardial infarction this mechanism is disrupted by the generation of reactive oxygen species (ROS) such as superoxide, leading to a marked loss of COX activity [40]. These results are consistent with the well-established concept that suppression of mitochondrial energy metabolism can lead to depression of cardiac contractile function [41].

The Conserved network was useful in the delineation of the cardiac response to increased protein synthesis and energy deprivation through activation of autophagy. This is a highly conserved cellular pro-survival mechanism for bulk lysosomal degradation of cytoplasmic components that mobilizes energy resources in response to starvation or hypoxia [42]. Autophagy also has a protein quality-control housekeeping function. The Conserved network identified two key genes related to autophagic processes, Atg5 (Autophagy-related protein 5) and Becn1 (Beclin-1). Both of these genes were topologically central to the Conserved network (betweenness centrality of 53356.0 and 12262.3 respectively), implicating them in critical mediation of network information flow. Recent studies in mice with temporally controlled cardiac-specific deficiency of Atg5 demonstrated that Atg5 was essential for normal physiological growth and function of the heart. However, Atg5-deficient animals developed contractile dysfunction and heart failure accompanied by increased levels of ubiquitinated proteins. Furthermore, Atg5-deficient hearts showed disorganized sarcomere structure and mitochondrial misalignment and aggregation [43]. These abnormalities were suggested, at least in part, to be due to loss of the protein quality control function of autophagy. Becn1 is part of a PI3K complex that plays an important role during the initiation of autophagosome formation [42,44]. Interestingly, mice with heterozygous disruption of Becn1 (Becn1^+/-^) exhibited reduced levels of autophagy during reperfusion but had decreased apoptosis and reduced infarct size compared to wild type mice [45], suggesting that in this case autophagy was detrimental. However, Becn1 is an important point of crosstalk with apoptotic pathways through its interaction with anti-apoptotic proteins such as Bcl-2 [46]. Disruption of Becn1 could therefore have pro- or anti-survival effects [42]. Of note, in the Conserved network, Becn1 localized to the same MCL cluster as Bcl-2, which is known to inhibit Becn1-depended autophagy [46]. Thus, in physiological LVH, autophagy compatible with cell survival, rather than cell death, may be regulated by coordinated changes in Atg5, Becn1 and Bcl-2. Indeed, autophagy- and proteolysis-related genes localized to the same cluster as genes involved in cell cycle regulation, providing further support for this hypothesis.

To explore if key regulatory mechanisms may be encoded by topologically significant nodes, the Conserved network was studied using concepts of betweenness centrality and node degree. These approaches are known to detect essential hubs in interaction networks [47] and previous studies have demonstrated that betweenness is a good indicator of biological essentiality [48]. Interestingly, when the top 200 hub genes were systematically removed from the Conserved network, average network betweenness remained mostly constant and high, while characteristic path length increased dramatically, to a threshold beyond which the network collapsed. This may suggest a presence of a large number of well-connected genes that preserve network information flow, possibly an indicator of maintained functional cardiac integrity during physiological remodeling. Additionally, topologically-central genes (core genes) localized to KEGG pathways including 'Oxidative phosphorylation' (*n *= 19 genes), 'MAPK signaling pathway' (*n *= 18 genes), and 'Focal adhesion' (*n *= 17 genes) (Figure 4E).

Several genes associated with the mammalian target of rapamycin (mTOR) pathway (Cab39, Hif1a, Tsc2) were also identified. The mTOR pathway controls changes in cell size following activation of the PI3K/Akt system. Akt phosphorylates the Tsc2 gene product tuberin, and thereby reduces its ability to stimulate GTP hydrolysis on the Ras-like G protein Rheb, leading to increased protein synthesis via ribosome biogenesis - a key feature of cardiac hypertrophy - and cell growth [49]. Recently, inhibition of the mTOR pathway by rapamycin was demonstrated to alleviate load-induced cardiac hypertrophy in mice, making it a potential therapeutic target [50]. Indeed, Tsc2 had a very large betweenness centrality value (174802.9, top 1%), confirming that it is one of the key constituents of the Conserved network. Core genes present in the 'MAPK signaling pathway' included Map4k3, Map3k7, Rap1a, Mapkapk2, Cacng2, and Ppm1b. Of these, Ppm1b (protein phosphatase 1B) had the greatest node degree (32) and betweenness centrality (73822.0) values, supporting its biological importance. These findings are reinforced by demonstration of direct inhibition of Map3k7 by Ppm1b [51], thus providing further evidence that Map3k7 activity is reduced in physiological hypertrophy protecting the heart from interstitial fibrosis, severe myocardial dysfunction, and apoptosis [52].

Similarly, the core Conserved network suggests that the genes involved in KEGG 'Calcium signaling pathway' may be involved in physiological LVH. There were 13 genes (e.g. Ppp3ca, Egfr, Vdac3, Slc25a4, Tnnc1) allocated to 'Calcium signaling pathway', of which Ppp3ca (calcineurin A alpha) had the largest betweenness centrality value (71043.2). Ppp3ca has been shown to be a key regulator of cardiac hypertrophy through activation of the transcription factor NFAT (nuclear factor of activated T-cells) which promotes the expression of pro-hypertrophic genes in concert with other transcription factors such as GATA4 and MEF2 [53]. It can also inhibit Map3k7 signaling [54]. The Conserved network also provides further evidence that calcineurin activity is highly regulated under physiological conditions by elucidation of the Rcn2 gene, which is known to inhibit calcineurin signaling [55].

The use of MCL in the core network (Figure 5) identified enriched clusters of genes participating in similar biological pathways. For example, cluster 1 was enriched for KEGG pathway 'Apoptosis' (*n *= 5 genes: Birc2, Irak1, Pik3ca, Prkaca, Ppp3ca). Birc2 (baculoviral IAP repeat-containing 2, betweenness = 3316.0) encodes a protein that inhibits apoptosis by binding to tumor necrosis factor receptor-associated factors TRAF1 and TRAF2. Although previously not reported in the mammalian heart, Birc2 was confirmed as a critical regulator of vascular integrity and endothelial cell survival in zebrafish [56]. Null mutants for Birc2 showed severe hemorrhage and vascular regression due to endothelial cell integrity defects and activation of Caspase-8-dependent apoptosis program. Coordinated regulation of angiogenesis is essential for preserved cardiac contractile function [57] and our results provide further molecular evidence for angiogenic gene programs in physiological LVH that merits further exploration.

## Conclusions

This report presents the first integrative analysis of genome-wide expression data and computational network inference in the context of physiological LVH. The identification of several mechanisms already known to be involved in physiological cardiac remodeling based on prior experimental studies provides confirmation to the validity of the approaches used in this study. In addition to supporting current molecular understanding of the cardiac physiological response to stress, this work characterizes topological and functional properties of 2128 potential molecular targets involved in the systematic regulation of physiological LVH. Additionally, we demonstrate for the first time the evolutionary complexity of the hypertrophic response. Our study suggests that evaluation of higher order relationships between genes and their neighbors, rather than mere individual over- or under-expression, may facilitate a better understanding of function in physiological and pathological phenotypes. Overall, the results offer new support for the utility of co-expression network modeling and the quality of public microarray data in the context of cardiac hypertrophy, facilitating further analysis of complex physiological and pathological phenotypes.

## Methods

### Data Preparation

Three publicly available mouse microarray datasets were included in this study, corresponding to 51 arrays. Individual mouse phenotypes under experimental conditions were reviewed carefully to ensure that each met physiological inclusion criteria (LVH with preserved or improved heart function and corresponding normal controls). Raw expression values were obtained from ArrayExpress database [58] and normalized using Robust Multi-array Average (RMA) [59]. Probesets with very low expression across experiments were removed and, in cases where multiple probesets mapped to a single gene, only those genes with the highest intensities were retained. To standardize annotation across multiple microarray platforms, Affymetrix probe identifiers were mapped to their corresponding Ensembl (August 12, 2009) gene identifiers (IDs) [60].

Pairwise similarity in gene expression vectors was expressed by the Pearson correlation coefficient (PCC). Gene pairs that correlated above a predefined PCC threshold value were represented in the form of an undirected unweighted network, where nodes (vertices) correspond to genes and links (edges) correspond to co-expression between genes. Randomized networks were generated by rewiring edges in the original networks while preserving the degrees of the respective nodes [21]. The number of rewiring steps taken for each model was 4× (number of edges). This method ensures that topological structure of the network is retained during randomization.

### Network consensus and topological analysis

A co-expression link between two genes was considered as a 'consensus' link, if it was observed in all three datasets. Topological properties examined were node degree, network diameter, betweenness centrality, connected components, clustering coefficient, and characteristic path length [61]. Node degree is defined as the total number of edges that connect to a given node. Network diameter is defined as the average shortest path between any pair of nodes in the network. Betweenness centrality is the measure of node importance within a graph, where nodes that occur on many shortest paths between nodes have higher betweenness. Connected components are maximal connected subgraphs of an undirected graph in which any two vertices are connected to each other by edges. Clustering coefficient is the degree to which nodes tend to cluster together. Characteristic path length is the average distance between pairs of vertices.

### Cluster Analysis and Functional Enrichment

Significant clusters of genes in a co-expression network were identified using Markov Cluster Algorithm (MCL) [62]. This is an efficient, unsupervised, and accurate graph clustering approach based on simulation of stochastic flow in graphs. To ensure significance of enrichment, only resulting clusters with 10 or more genes were further retained. A distinct advantage of MCL is its ability to avoid incorrect clustering assignments in the presence of false negative edges [6,62]. This is due to the fact that MCL discovers clusters by virtue of genes sharing higher-order connectivity in their local neighborhoods and not merely pairwise linkages. Genes identified to be present in the same cluster were analyzed for overrepresented (enriched) Gene Ontology Biological Process (GO-BP) terms and KEGG pathways [22] using the log-odds ratio. Higher ratio indicates a higher relative abundance of a GO-BP term or KEGG pathway in a cluster compared to the entire network. While all KEGG pathways were considered for enrichment, to avoid broad annotation terms, only GO-BP categories with fewer than 1,500 genes (mouse annotations) were retained [63].

### Evolutionary gene analysis

Evolutionary conservation was computed by comparing selected protein sequences from the core network (corresponding to 1020 genes) against the complete genomes of 287 species available in the Complete Genome Tracking (COGENT) database [64] database using BLAST [65] with default parameters. Significant hits from this run have been retained with a p-value cut-off < 10^-06^, corresponding to 100532 pairwise similarity relationships. Homology networks were visualized using the Large Graph Layout (LGL) software [66].

## Authors' contributions

ID performed data integration, statistical and network analysis and drafted the initial manuscript. ST has contributed to the analysis and the manuscript. CAO carried out computational analysis, edited the manuscript and coordinated the research. AMS coordinated the project design and edited the manuscript. All authors read and approved the manuscript.

## Supplementary Material

Additional file 1 **Table of Conserved network genes in physiological cardiac hypertrophy (sorted by node degree)**. Click here for file

Additional file 2 **Gene edge list for the Conserved physiological cardiac hypertrophy network**. Click here for file

Additional file 3 **An edge list for 100532 pairwise sequence similarities covering 64550 unique homologues**. Click here for file

Additional file 4 **Specificity of the Conserved network genes to the current knowledge of cardiac biology**. **(A) **933/2128 (44%) genes in the Conserved network had at least one abstract per PubMed search terms 'Hypertrophy', 'Heart', or 'Heart Failure'. Number of abstracts per gene is reflected both by a color scale blue-red and node size. **(B) **PubMed abstract frequency histogram of 933 genes confirmed to be related to cardiac biology. Click here for file
