# Supplementary figures and images for: Genome-wide expression patterns in physiological cardiac hypertrophy
Source: BMC Genomics. 2010 Oct 11;11:557. doi: 10.1186/1471-2164-11-557 (PMC3091706; doi:10.1186/1471-2164-11-557)

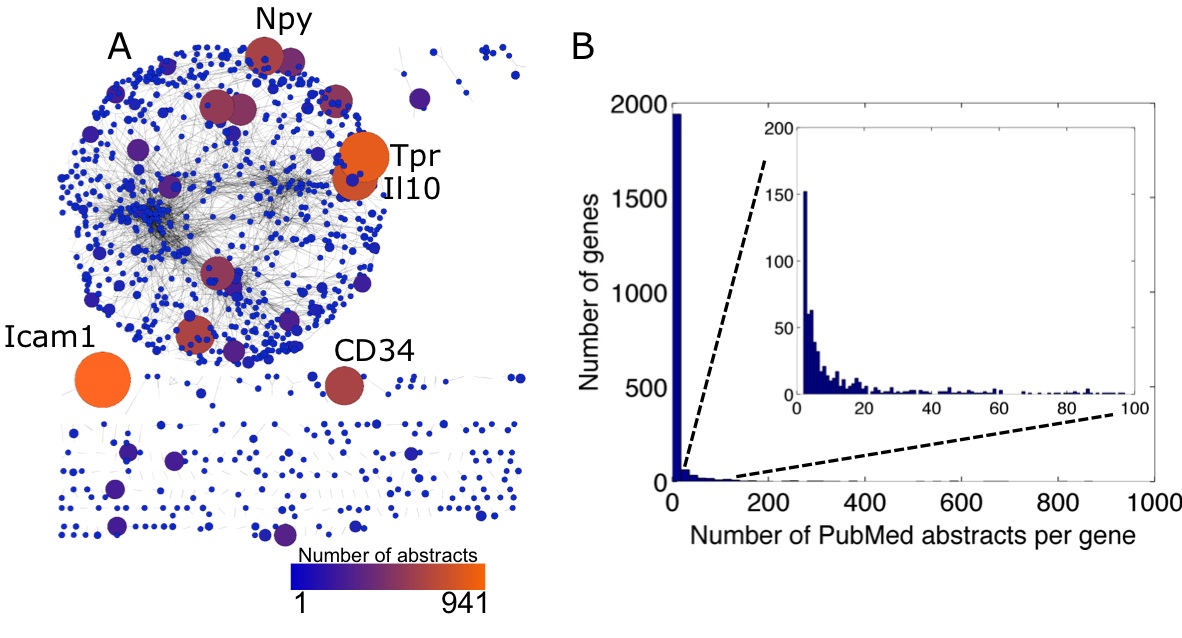

Supplement: Additional file 4 — Specificity of the Conserved network genes to the current knowledge of cardiac biology. (A) 933/2128 (44%) genes in the Conserved network had at least one abstract per PubMed search terms 'Hypertrophy', 'Heart', or 'Heart Failure'. Number of abstracts per gene is reflected both by a color scale blue-red and node size. (B) PubMed abstract frequency histogram of 933 genes confirmed to be related to cardiac biology. [file 1471-2164-11-557-S4.JPEG]
